# Intranasal Dexmedetomidine Accompanied by Cartoon Video Preoperation for Reducing Emergence Delirium in Children Undergoing Strabismus Surgery: A Prospective Randomized Trial

**DOI:** 10.3389/fsurg.2021.754591

**Published:** 2021-10-22

**Authors:** Liyan Chu, Yue Wang, Shanshan Wang, Shaofei Su, Zhixing Guo, Guyan Wang

**Affiliations:** ^1^Department of Anesthesiology, Beijing Tongren Hospital, Capital Medical University, Beijing, China; ^2^Central Laboratory, Beijing Obstetrics and Gynecology Hospital, Capital Medical University, Beijing, China; ^3^Department of Anesthesiology, National Cancer Center/National Clinical Research Center for Cancer/Cancer Hospital, Chinese Academy of Medical Sciences and Peking Union Medical College, Beijing, China

**Keywords:** dexmedetomidine, emergence delirium, video, children, preschool, intranasal

## Abstract

**Background:** After general anesthesia, many pediatric patients present with emergence delirium (ED). The aim of this study was to determine whether dexmedetomidine intranasal premedication accompanied by a cartoon video 30 min before general anesthesia would have an effect on reducing emergence delirium in preschool children.

**Methods:** One hundred and forty children aged 3–6 year undergoing elective strabismus surgery were randomly to be premedicated with 2 μg kg^−1^ intranasal dexmedetomidine accompanied by the viewing of a cartoon video (Group DV) or without any premedication as usual (Group C). The primary outcome was the incidence of emergence delirium at the postanesthesia care unit (PACU), evaluated by the Pediatric Anesthesia Emergence Delirium (PAED) scale. The secondary outcomes included: the Modified Yale Preoperative Anxiety Scale (mYPAS) upon separation from parents; the Induction Compliance Checklist score (ICC); the PACU discharge time; the parental satisfaction score; the incidences of the side effects and the Post-Hospital Behavior Questionnaire (PHBQ) score during the first day after surgery.

**Results:** The incidence of emergence agitation (PAED score ≥ 10) was reduced in Group DV compared with Group C [8 (11.4%) vs. 24 (34.3%); *P* = 0.001]. None of the patients in the DV group experienced severe emergence agitation (PAED score ≥ 15), as compared with the C group (*P* = 0.006). The mYPAS score upon separation from parents (*P* < 0.001) and the incidence of poor coordination (ICC ≥ 4) during induction (*P* < 0.001) were significantly lower in Group DV than in Group C. In Group DV, the PACU discharge time was longer (*P* < 0.001), and the parental satisfaction score was higher (*P* < 0.001). However, during the first day after surgery, the PHBQ score was lower in Group DV compared with Group C (*P* = 0.001).

**Conclusions:** Premedication with 2 μg kg^−1^ intranasal dexmedetomidine accompanied by cartoon video viewing can dramatically reduce emergence delirium in preschool children undergoing strabismus surgery, relieve preoperative anxiety and improve the parental satisfaction and the postoperative behavior changes during the first day after surgery.

**Clinical Trial Registration:** ChiCTR2000030678.

## Introduction

Strabismus surgery is a typical pediatric procedure that involves general anesthesia. After strabismus surgery, many pediatric patients present with emergence delirium (ED), and the incidence rates are as high as 40–86% (1). ED can lead to various adverse effects, such as increasing risks of suture dehiscence or accidental removal of intravenous catheters, self-injury, prolonged postanesthesia care unit (PACU) length of stay, and even negative postoperative behavior (2, 3). However, the pathogenesis of postoperative ED remains unclear (4). Many risk factors are associated with ED, such as preschool age, preoperative anxiety, pain, otorhinolaryngologic and ophthalmic surgery, some anesthetic agents, rapid emergence from anesthesia (5), and most of them are closely related to strabismus surgery. And in our hospital, a large number of strabismus surgeries in preschool children are performed every day. To save time, the children are transported to the holding area approximately 30 min before anesthesia for preparation and then enter the operating room, separating from their parents. All of the above make the children become anxious. As it was reported that significant preoperative anxiety affected up to 60% of young children undergoing anesthesia and surgery, and high preoperative anxiety levels led to ED (6, 7). Various pharmacological agents have been used to reduce the incidence of ED (8–11). Dexmedetomidine is a highly selective α-2 adrenergic agonist with sedative and analgesic effects (10). Intranasal dexmedetomidine has a slower and more gradual onset than intravenous administration (12), with a lower incidence of nasal irritation (13); Abdel-Ghaffar's study revealed that premedication with nebulized 2 μg kg^−1^ dexmedetomidine resulted in less postoperative agitation in preschool children. But some of the children still could not cooperate well during administration, although there was no pain and no bad taste during the intranasal premedication (10). On the other hand, some non-pharmaceutical methods seem popular to the preschool children, such as parental presence, cartoon distraction, pediatric anesthesia comic information leaflet, transportation by a children's ride-on car, the preoperative preparation workshops and the mother's recorded voice (3, 14–18). Nevertheless, most of them are restricted by space and staff, and are not suitable for a large number surgeries. Viewing the cartoon video seems convenient and attractive for preschooler.

Therefore, we hypothesized that premedication with intranasal dexmedetomidine accompanied by a cartoon video would reduce preoperative anxiety and ED. The aim of this study was to determine the impact of premedication with intranasal dexmedetomidine accompanied by a cartoon video on the ED of preschool children undergoing strabismus surgery.

## Materials and Methods

### Enrollment and Eligibility

Following approval by the biomedicine ethics committee of Beijing Tongren Hospital (TRECKY2019-072), this prospective randomized study was registered at the Chinese Clinical Trial Registry (ChiCTR2000030678) on March 9, 2020. Then, it was conducted in Beijing Tongren Hospital from April 2020 to October 2020, the final study population was 140 patients.

All patients aged 3–6 yr, with American Society Anesthesiologists (ASA) grade I or II, hospitalized in Beijing Tongren Hospital and undergoing their first elective strabismus surgery, were candidates to participate in this study. Written informed consent was obtained from the parents or authorized guardian representatives before participation in the study. Patients with each one of following conditions were excluded from the study: (1) a history of neurological and psychiatric disease; (2) use of analgesic and sedative drugs; (3) body mass index > 20 kg m^−2^; (4) important organ comorbidities; (5) mentally retarded children; (6) allergy to dexmedetomidine.

### Randomization and Blinding

According to a computer-generated randomization table, 140 patients were assigned to two groups that either rested with their parents without any premedication (Group C, 70 patients), or received premedication of intranasal 2 μg kg^−1^ dexmedetomidine and began to watch a cartoon video by a unified eye-protection mode 10 min before separation from their parents until anesthesia induced sleep (Group DV, 70 patients). The group allocation was concealed in sealed opaque envelopes. An independent nurse not involved in the study opened a sealed envelope the day before surgery.

The anesthetist in charge of evaluating the PAED score, the recovery condition and follow-up visit the next day was specially trained and blinded to the allocation.

## Methods

The day before surgery, the eligible patients were visited by a trained anesthetist, and their general information and temperaments were recorded. The patients in the DV group were allowed to choose their favorite cartoon videos with their parents for the next day. All of them were fasted according to the guidelines: 8 h for any solid food, 6 h for milk, and 2 h for clear liquid. All of the patients were brought to the holding area 30 min before the surgery and treated according to the group assignment. All of the patients were transported on a gurney.

After the application of standard monitoring, including blood pressure, electrocardiography, capnography, and the bispectral index, general anesthesia was started by tidal volume inhalation induction with 8% sevoflurane and 5 L/min 100% oxygen. At the same time, the Induction Compliance Checklist (ICC) score was determined by the trained nurse. IV access was obtained, and an appropriately sized laryngeal tube was inserted when a suitable anesthetic depth was obtained. Then, 0.02 mg kg^−1^ atropine and 0.1 μg kg^−1^ sufentanil were administered, and the sevoflurane concentration was adjusted to 2–4% to maintain anesthesia. Spontaneous breathing was maintained during surgery. If the P_ET_CO_2_ was more than 50 mmHg, assisted ventilation was conducted. At the end of the surgery, the anesthetist stopped administering sevoflurane, removed the laryngeal mask when necessary and sent the patient to the PACU for observation.

## Assessment Parameters

The modified Yale Preoperative Anxiety Scale (mYPAS) was applied by the trained anesthetist to evaluate anxiety at the moment each child was separated from his or her parents. The mYPAS score was used to evaluate the children's anxiety, including 27 items in five behavioral categories (activity, emotional expressivity, state of arousal, vocalization, and use of parents) (19). The score ranges from 22.9 to 100, and a score ≥ 40 indicates the presence of anxiety.

The ICC score was applied to evaluate the induction time. It was used to assess cooperation of the child for mask holding. A scoring system on a 0–10 scale was provided by Kain et al., (20) where a perfect induction (in which the child does not exhibit negative behaviors, fear, or anxiety) is scored as “0” and the case of a child with fear, negative behavior, and anxiety is scored as “10.” A validated simplified 3-point scoring system for ICC by Varughese et al., (21) [perfect (ICC = 0), moderate (ICC = 1–3), and poor (ICC ≥ 4)] was used in our trial during induction.

The Hindi version of the State-Trait Anxiety Inventory (STAI) scale was used to evaluate parental anxiety on the day of surgery. The STAI is a self-report anxiety assessment instrument containing two separate, 20-item rating scales for measuring trait and state anxiety (22, 23). Total scores for state and trait anxiety range from 20 to 80 each; higher scores denote higher levels of anxiety.

A specially trained anesthetist blinded to the study assessed the Pediatric Anesthesia Emergence Delirium (PAED) scale every 10 min during the recovery period until the time children departed from the PACU. The scale was used to measure the severity of the children's agitation in the recovery room (24). A threshold score of 10 was considered a discriminator of the presence or absence of agitation and for the need for treatment. A score ≥ 15 signified severe ED, and 1 mg kg^−1^ propofol was given for rescue agitation in this study. The PACU discharge time and the incidences of side effects were also collected.

One day after surgery, the special anesthetist followed up with the patient and recorded the parental satisfaction score and adverse reactions such as pain, nausea, swirling, drowsiness in the inpatient ward and nervousness or crying at the follow-up visit. The Post-Hospital Behavior Questionnaire (PHBQ) score was also completed by the parent. The PHBQ score contains a 27-item measure of negative postoperative behavior changes (i.e., anxiety, regression, eating disturbance, and aggression) (25), compared with baseline. A PHBQ score>0 signifies a negative postoperative behavioral change.

## Statistical Analysis

### Power of the Study

The primary outcome of the study was the incidence of ED. The secondary outcomes were the modified Yale Preoperative Anxiety Scale (mYPAS) of the children; the STAI scale of the parents; the ICC score; the PACU discharge time; the parental satisfaction score; the incidences of side effects; and the PHBQ score the first day after surgery.

Based on our pilot study, the mean (SD) incidence of ED in the control group was 30.0%, and 59 patients in each group were sufficient to detect a reduction in 20%, with a power (1-β) of 80% and a two-sided-type I error of 5%. The sample size for two groups was enlarged to 140 to allow for attrition.

### Data Analysis

All the data were analyzed by the SAS 9.4 statistical analysis system. Quantitative variables with a normal distribution are expressed as the mean (standard deviation), and the independent sample *t*-test was used for comparisons among groups. Categorical variables were statistically described by the number of cases (percentage %), and the chi-square test or Fisher's exact probability method was used for comparisons among groups. All tests were two-sided tests, and *P* < 0.05 was considered statistically significant.

## Results

**Figure 1:**
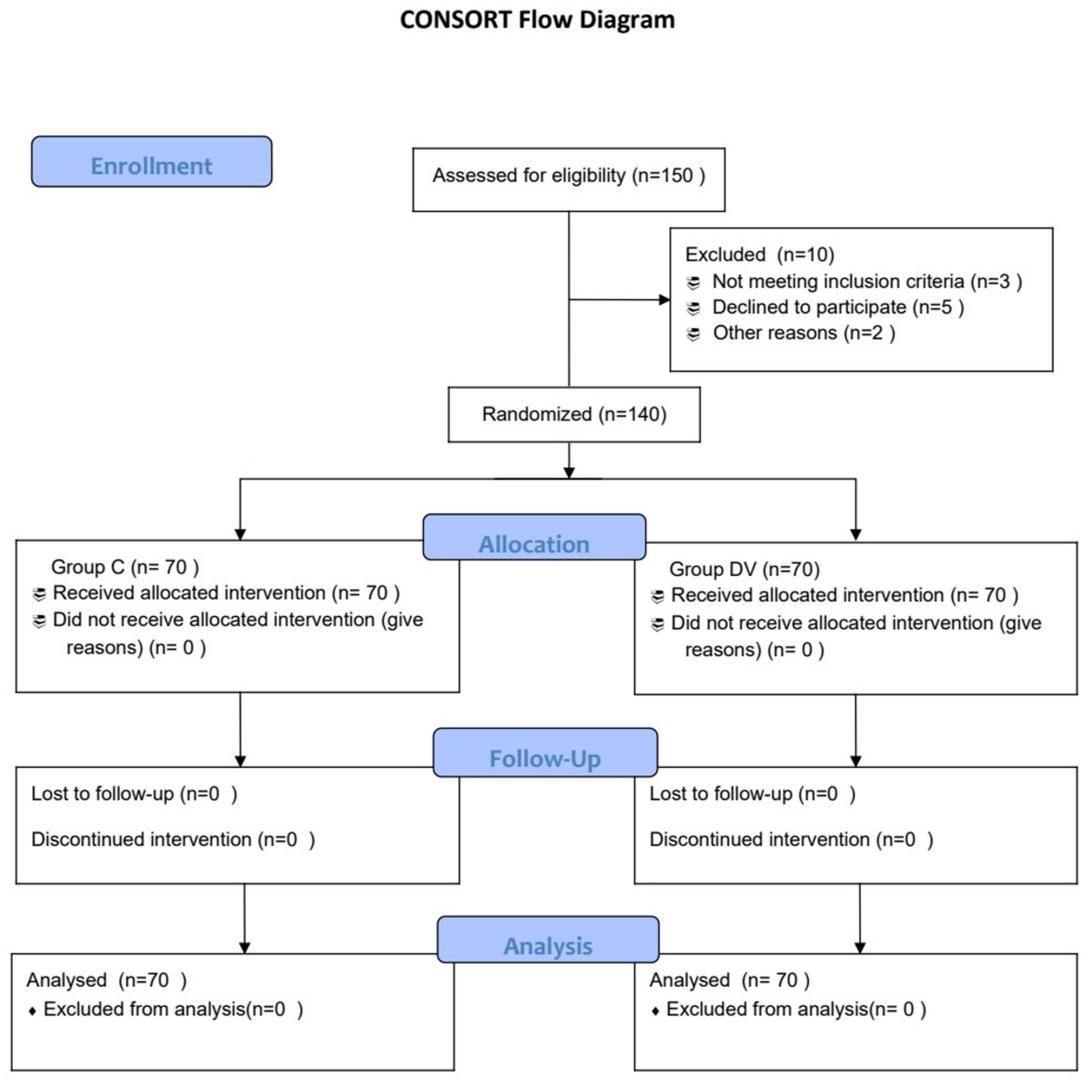
Consort flow diagram.

As shown in the Consort flow chart (Figure 1), 150 children were assessed for eligibility from April 2020 to October 2020, and 140 patients were enrolled in our study and then randomized into one of two groups (*n* = 70). There were no significant differences between the groups in terms of age, sex, body mass index (BMI), the ratio of one-eyed surgery, or the parental STAI score (Table 1).

**Table 1:** Subject characteristics and clinical data.

**Variables**	**Group C (*n* = 70)**	**Group DV (*n* = 70)**	**Statistic**	***P*-value**
Age(yr)^a^	4.7 (1.0)	4.73 (1.0)	0.2	0.864
BMI(kg.m^2^)^a^	15.9 (1.5)	15.9 (1.6)	0.2	0.850
Sex(Male) *n* (%)^b^	36 (51.4)	31 (44.3)	0.7	0.398
Single eye *n* (%)^b^	34 (48.6)	33 (47.1)	0.1	0.866
m-YPAS score separating from parents^a^	44.5 (17.9)	27.8 (9.8)^∧^	6.9	<0.001
Incidence of m-YPAS score ≥ 40 *n* (%)^b^	40 (57.1)	8 (11.4)^∧^	32.5	<0.001
ICC score^a^	3.8 (3.1)	1.0 (1.7)^∧^	6.5	<0.001
Incidence of ICC ≥ 4 *n* (%)^b^	29 (41.4)	7 (10.0)^∧^	18.1	<0.001
STAI score^a^	43.8 (8.7)	42.43 (8.4)	0.8	0.438
STAI-特质焦虑^a^	38.6 (6.0)	40.84 (8.2)	1.6	0.112

*Data are described as mean (standard deviation) or number (frequency). m-YPAS score, the modified Yale Preoperative Anxiety Scale; ICC, the Induction Compliance Checklist; STAI, the State-Trait Anxiety Inventory. Group DV (intranasal 2ug kg^−1^ dexmedetomidine accompanied by cartoon video), Group C (control group)*.

a *t-Test for independent samples*.

b *the chi-square test was used. two-tailed, P < 0.05 is significant*.

∧ *P < 0.001*.

## Emergence Agitation

The incidence of emergence agitation (PAED score ≥ 10) in the DV group was reduced dramatically compared with that in the other group [8 (11.4%) vs. 24 (34.3%); *P* = 0.001; Table 2]. In addition, 8 (11.4%) of the patients in the control group experienced severe emergence agitation (PAED score ≥ 15), in contrast to 0 (0.0%) in the DV group (*P* = 0.006; Table 2). Moreover, the maximal PAED score of the DV group was significantly lower than that of the C group [4.2 (3.2) vs. 7.7 (4.5); *P* < 0.001; Table 2].

**Table 2:** Postoperation situation in PACU.

**Variables**	**Group C (*n* = 70)**	**Group DV (*n* = 70)**	**Statistic**	***P*-value**
PAED score^a^	7.7 (4.5)	4.2 (3.2) ^∧^	5.3	<0.001
incidence of ED (PAED ≥ 10) *n* (%)^b^	24 (34.3)	8 (11.4)^$^	10.4	0.001
incidence of serve ED (PAED ≥ 15) *n* (%) ^c^	8 (11.4)	0 (0.0)^$^	–	0.006
PACU discharge time (min)^a^	25.0 (10.9)	34.5 (12.2)^∧^	4.9	<0.001

*Data are described as mean (standard deviation) or number (frequency). PAED, Pediatric Anesthesia Emergence Delirium scale. Group DV (intranasal 2ug kg^−1^ dexmedetomidine accompanied by cartoon video), Group C (control group)*.

a *t-Test for independent samples*.

b *the chi-square test or*

c *Fisher's exact probability method was used. two-tailed, P < 0.05 is significant*.

$ *P < 0.01*,

∧ *P < 0.001*.

The PACU discharge time of the DV group was significantly different from that of the C group [34.5 (12.2) min vs. 25.0 (10.9) min, *P* < 0.001] (Table 2).

## Preoperative Anxiety

When separated from the parents, the mYPAS score of the DV group was obviously lower than that of the C group [27.8 (9.8) vs. 44.5 (17.9), *P* < 0.001] (Table 2). The ICC score of the C group was significantly higher than that of the DV group (*P* < 0.001; Table 2). The incidence of ICC ≥ 4 in the C group was significantly higher than that in the DV group [29 (41.4%) vs. 7 (10.0%), *P* < 0.001] (Table 2).

## Side Effects, Parental Satisfaction Score and PHBQ Score on the Day After Surgery

Fifteen patients in group C and 1 in group DV displayed nervousness (*P* = 0.001; Table 3); 15 patients in group C and 4 in group DV exhibited crying (*P* = 0.007; Table 3). Four patients in group C and 3 in group DV experienced nausea (*P* = 0.698; Table 3). The incidences of drowsiness and swirl in the C group were the same as those in the DV group (*P* = 1.000; Table 3). The parental satisfaction score of the DV group was 9.6 (0.8), which was significantly higher than that of the C group [8.5 (1.2), *P* < 0.001] (Table 3). During the first day after surgery, the PHBQ score of the C group was significantly higher than that of the DV group (*P* = 0.001; Table 3).

**Table 3:** Side-effects and the parental satisfaction score and the PHBQ score.

**Variables**	**Group C *n* (%)(*n* = 70)**	**Group DV *n* (%)(*n* = 70)**	**Statistic**	***P*-value**
Nervous^b^	15 (21.4)	1 (1.4)^$^	13.8	0.001
Cry^b^	15 (21.4)	4 (5.7)^$^	7.4	0.007
Drowsiness^c^	1 (1.4)	1 (1.4)	–	1.000
Nausea^c^	4 (5.7)	3 (4.3)	–	0.698
Swirl^c^	1 (1.4)	1 (1.4)	–	1.000
Parental satisfaction score^a^	8.5 (1.2)	9.6 (0.8)^∧^	6.0	<0.001
The PHBQ of one day after surgery^a^	3.4 (2.8)	1.4 (1.8)^$^	4.9	0.001

*PHBQ, the Post-Hospital Behavior Questionnaire score of the first day after surgery. Data are described as mean (standard deviation) or number (frequency). Group DV (intranasal 2ug kg^−1^ dexmedetomidine accompanied by cartoon video), Group C (control group)*.

a *t-Test for independent samples*.

b *the chi-square test or*

c *Fisher's exact probability method was used. two-tailed, P < 0.05 is significant*.

$ *P < 0.01*,

∧ *P < 0.001*.

## Discussions

This study showed that for children undergoing strabismus surgery, premedication with intranasal 2 μg kg^−1^ dexmedetomidine accompanied by a cartoon video significantly reduced the incidence of emergence delirium, improved the postoperative behavior changes during the first day after surgery and the parental satisfaction score. Moreover, the children in the DV group showed lower anxiety when separating from their parents and more cooperation when anesthesia induction.

In contrast with general surgeries, strabismus surgery is not very traumatic, but the incidence of emergence agitation is still high, not only due to the anesthesia course mainly consisting of sevoflurane and the fear of dressing coverage but also because of the distressing preoperative period. Separation from parents, unfamiliar environments, fear of surgery, and venipuncture can cause children to cry and struggle. In fact, the preoperative period is distressing for preschool children (8). The higher mYPAS score and ICC score in the control group in this study proved the presence of serious anxiety in children receiving no intervention. Different from Abdel-Ghaffar's study (10), when the premedication was accompanied by viewing a cartoon video, the preschool patients in the DV group felt more relaxed and had more satisfactory experience. It was proven by the lower mYPAS score upon separation from the parents and the ICC score during anesthesia induction.

In the DV group, the incidence of ED was 22.9% lower than the control group, even though none of the children suffered from severe ED, not only due to the lower preoperative anxiety, but also because of the continuous function of dexmedetomidine. Dexmedetomidine acts on receptors in the locus coeruleus of the pons, providing sedation, and exerts dose-dependent moderate primary analgesic effects through activation of α-2 adrenoreceptors in the dorsal spinal horn, causing a subsequent decrease in substance P release (26). Its median time to reach peak concentration was 37 min (27), which is appropriate to the preoperative time. Dexmedetomidine can relieve pain and calm patients for a period of time, when sevoflurane metabolizes stably and completely. Therefore, dexmedetomidine is used to alleviate sevoflurane-induced ED in children (28). Intranasal dexmedetomidine accompanied by watching a cartoon video not only relieved the patients' preoperative anxiety but also improved their recovery quality.

Since our anesthesia measure was different from that in Yusheng Yao's study (29), we did not find a lower incidence of nausea in the DV group. Fifteen patients in the C group appeared nervous or cried when the medical staff interviewed them on the day after surgery. In contrast to this phenomenon, the DV group patients behaved more naturally, even when some of them underwent the course of premedication and induction by themselves. The lower anxiety and sedation induced by dexmedetomidine might have contributed to this difference between the two groups. Moreover, this experience also led to a higher satisfaction score of the parents in the DV group.

It has been reported that on the first day after sevoflurane anesthesia, the incidence of negative postoperative behavioral changes is 60% and that dexmedetomidine may be effective in reducing the incidence of postoperative behavior changes (30, 31). As shown in this study, the PHBQ score on the first day was lower in the DV group than in the control group. Because dexmedetomidine has a sedative effect mediated by binding to postsynaptic α-2 receptors in the locus coeruleus, reducing noradrenergic output and thereby facilitating the firing of inhibitory neurons, and those in the g-aminobutyric acid system. Its analgesic action functions through its binding to α-2 receptors in the dorsal horn and supra-spinal sites and thereby reducing the release of substance P (32). However, the incidence of postoperative behavior changes in the DV group was still 37.14% because despite the presence of anxiety, many other factors, such as temperament and postsurgical pain, might contribute to postoperative behavior changes (33, 34).

Notably, it is reported that dexmedetomidine does not extend the PACU stay duration obviously, (1) but in this study, the average PACU discharge time of the DV group was much longer. This may have been caused by different anesthesia methods; thus, more research is needed to improve it.

## Limitations

The limitation of this study is that we only focused on preschool children and sevoflurane inhalation anesthesia. Therefore, more studies will be performed to investigate other age groups or other general anesthesia methods. The PACU discharge time was delayed in this study, and we need to find some measure that does not affect discharge.

## Conclusions

In conclusion, premedication with 2 μg kg ^−1^ intranasal dexmedetomidine accompanied by a cartoon video can dramatically reduce emergence delirium in preschool children undergoing strabismus surgery, relieve preoperative anxiety and improve the postoperative behavior changes during the first day after surgery and parental satisfaction.

## Data Availability Statement

The raw data supporting the conclusions of this article will be made available by the authors, without undue reservation.

## Ethics Statement

The studies involving human participants were reviewed and approved from the biomedicine Ethics Committee of Beijing Tongren Hospital (TRECKY2019-072). Written informed consent to participate in this study was provided by the participants' legal guardian/next of kin.

## Author Contributions

LC and GW designed this study and conducted it. LC, YW, and SW were responsible for the collection of data. LC, GW, and ZG took charge of writing and editing of the manuscript. LC, SS, ZG, and YW done the excel sheet revision and statistical analysis. All authors contributed to the article and approved the submitted version.

## Funding

This work was supported by the Beijing Hospitals Authority Clinical Medicine Development of Special Funding Support (Grant No. ZYLX202103). The funders had no role in study design, data collection and analysis, decision to publish, or preparation of the manuscript.

## Conflict of Interest

The authors declare that the research was conducted in the absence of any commercial or financial relationships that could be construed as a potential conflict of interest.

## Publisher's Note

All claims expressed in this article are solely those of the authors and do not necessarily represent those of their affiliated organizations, or those of the publisher, the editors and the reviewers. Any product that may be evaluated in this article, or claim that may be made by its manufacturer, is not guaranteed or endorsed by the publisher.
